# Better haemodynamic stability under xenon anaesthesia than under isoflurane anaesthesia during partial nephrectomy – a secondary analysis of a randomised controlled trial

**DOI:** 10.1186/s12871-019-0799-2

**Published:** 2019-07-09

**Authors:** Patrick Schäfer, Astrid Fahlenkamp, Rolf Rossaint, Mark Coburn, Ana Kowark

**Affiliations:** 1 Department of Anaesthesiology, Medical Faculty RWTH Aachen University, Pauwelsstr. 30, 52074 Aachen, Germany; 2 Department of Anaesthesiology and Intensive Care Medicine, Campus Virchow-Klinikum, Charité–Universitätsmedizin Berlin, Berlin, Germany

**Keywords:** Xenon, Isoflurane, Haemodynamic stability, Renal function, Nephroprotection

## Abstract

**Background:**

Renal dysfunction following intraoperative arterial hypotension is mainly caused by an insufficient renal blood flow. It is associated with higher mortality and morbidity rates. We hypothesised that the intraoperative haemodynamics are more stable during xenon anaesthesia than during isoflurane anaesthesia in patients undergoing partial nephrectomy.

**Methods:**

We performed a secondary analysis of the haemodynamic variables collected during the randomised, single-blinded, single-centre PaNeX study, which analysed the postoperative renal function in 46 patients who underwent partial nephrectomy. The patients received either xenon or isoflurane anaesthesia with 1:1 allocation ratio. We analysed the duration of the intraoperative systolic blood pressure decrease by > 40% from baseline values and the cumulative duration of a mean arterial blood pressure (MAP) of < 65 mmHg as primary outcomes. The secondary outcomes were related to other blood pressure thresholds, the amount of administered norepinephrine, and the analysis of confounding factors on the haemodynamic stability.

**Results:**

The periods of an MAP of < 65 mmHg were significantly shorter in the xenon group than in the isoflurane group. The medians [interquartile range] were 0 [0–10.0] and 25.0 [10.0–47.5] minutes, for the xenon and isoflurane group, respectively (*P* = 0.002). However, the cumulative duration of a systolic blood pressure decrease by > 40% did not significantly differ between the groups (*P* = 0.51). The periods with a systolic blood pressure decrease by 20% from baseline, MAP decrease to values < 60 mmHg, and the need for norepinephrine, as well as the cumulative dose of norepinephrine were significantly shorter and lower, respectively, in the xenon group. The confounding factors, such as demographic data, surgical technique, or anaesthesia data, were similar in the two groups.

**Conclusion:**

The patients undergoing xenon anaesthesia showed a better haemodynamic stability, which might be attributed to the xenon properties. The indirect effect of xenon anaesthesia might be of importance for the preservation of renal function during renal surgery and needs further elaboration.

**Trial registration:**

ClinicalTrials.gov: NCT01839084. Registered 24 April 2013.

**Electronic supplementary material:**

The online version of this article (10.1186/s12871-019-0799-2) contains supplementary material, which is available to authorized users.

## Introduction

The current “gold-standard” for curative surgery of renal cell carcinomas is partial nephrectomy [1]. Although efforts have been made to further reduce the iatrogenic tissue trauma during surgery in the last years, it remains unclear how the anaesthesia could have a lasting effect on the patients’ outcome. The intraoperative renal dysfunction frequently induces a postoperative renal insufficiency [2]. Thus, the preservation of the renal perfusion and function has a great value in anaesthesiological management [3]. The risk factors for renal dysfunction are intraoperative hypovolaemia and hypotension, nephrotoxic drugs, and cardiovascular and renal comorbidities [3–6]. The avoidance of intraoperative hypotension is of great importance, as it is not only associated with an increased risk for acute renal failure, but also with other serious adverse events, such as mortality, stroke, and acute myocardial damage [7–11]. The optimum blood pressure target thresholds have not been established yet [11, 12]. The German Society for Anaesthesiology and Intensive Medicine (DGAI) has recently published a recommendation for intraoperative blood pressure thresholds. An intraoperative decrease of the absolute mean arterial blood pressure (MAP) to less than 55–65 mmHg or a relative systolic blood pressure decrease of more than 40–50% of the baseline value should be avoided [13]. Even a brief, 5-min MAP decrease to values less than 55–65 mmHg is associated with a significantly higher incidence of acute kidney injury and myocardial infarction [9, 14]. The haemodynamic management includes the use of fluids, inotropic drugs [3], and the optimised choice of anaesthetics. An ideal anaesthetic should preserve the cardiovascular stability and exhibit nephroprotective properties. Such properties were found in xenon in animal models [15–21]. Thus far, renal impairment has not been observed after a xenon anaesthesia in humans [22–24]. Our group recently conducted the PaNeX-study, wherein we hypothesised that the postoperative renal function after partial nephrectomy would differ between patients undergoing xenon and those undergoing isoflurane anaesthesia [25]. The primary endpoint was the maximum postoperative decrease of the glomerular filtration rate (GFR) until the seventh postoperative day. The results indicated that xenon has a potential nephroprotective effect. The underlying mechanism remained unclear. The direct nephroprotective effect, as well as the indirect effect via more stable intraoperative haemodynamics were discussed. However, the haemodynamic variables were not analysed in detail; only the mean systolic and diastolic blood pressures were described. Significantly more adverse events (*P* = 0.001), in particular, a higher incidence of intraoperative hypotension requiring catecholamine therapy (*P* = 0.003), were found in the isoflurane group. The intraoperative hypotension was not predefined in the PaNeX study; rather, it was based on the attending anaesthetist’s judgement. Therefore, we have performed a predefined secondary analysis of the particular haemodynamic differences between the two study groups of the PaNeX study. We hypothesized that the patients undergoing xenon anaesthesia would exhibit significantly better haemodynamics during the surgery, as defined by the recommendation of the DGAI [13]. To the best of our knowledge, this is the first study that analysed in detail the haemodynamics of patients undergoing xenon anaesthesia.

## Methods

### Study design

This is a predefined secondary analysis of the prospective, single-blinded, single-centre, randomised controlled PaNeX trial [25], which was conducted between July 2013 and October 2015 in the University Hospital of the RWTH Aachen, in Aachen, Germany. The Ethics Committee of the Medical Faculty RWTH Aachen University approved the study in April 2013 (EK 012/13). Registration was performed with ClinicalTrials.gov (NCT01839084) and Eudra-CT (identifier: 2012–005698-30). The complete methodological details are presented elsewhere [25].

The objective of this analysis was to compare the haemodynamic stability under xenon anaesthesia with that under isoflurane anaesthesia and to explore its effects on the renal perfusion during partial nephrectomy. The potential indirect effects should be presented in greater detail.

### Participants

All 46 patients of the PaNeX study [25] diagnosed with renal cell carcinoma and scheduled for a unilateral partial nephrectomy were included in this secondary analysis. The study groups included adult patients with an American Society of Anesthesiologists (ASA) status ≤II, without pre-existing severe cardiac, respiratory, or neurological diseases, allergies or contraindications to the study drugs, or stage 3 chronic renal insufficiency (GFR < 60 ml min^− 1^ 1.73 m ^− 2^).

### Study conduction

A detailed description of the study is presented elsewhere [25]. In brief, the patients were randomly allocated 1:1 to receive xenon (*n* = 23) or isoflurane (n = 23) anaesthesia, following the placement of a thoracic epidural catheter and anaesthesia induction using propofol, sufentanil, and rocuronium. The target anaesthetic concentration was either 60% inspired xenon with 40% oxygen or 1.2% end-expiratory isoflurane with 40% oxygen. Further anaesthesia conduction was performed according to the clinical routine, at the discretion of the attending anaesthetist. Haemodynamic management was performed according to the standard operating procedure (SOP) for partial nephrectomy in our department; the goal was to maintain the MAP ≥65 mmHg by application of fluids and norepinephrine, as deemed clinically appropriate. Opioids were titrated according to the patients’ need. A bolus dose of 6–7 ml of the mixture of 0.3% ropivacaine and 0.75 μg ml^− 1^ sufentanil was applied into the epidural catheter before the skin incision, followed by a continuous application during the surgery. Only the patients were blinded during the study.

### Outcome measures

All data used in this secondary analysis were prospectively collected. The blood pressure was measured every 5 min using non-invasive monitoring in all patients. Each measured value at these 5-min time-points was assumed to be representative for the preceding 5-min time span.

The primary outcome was the haemodynamic stability during anaesthesia, assessed by two analyses. First, the duration of a systolic blood pressure decrease by more than 40% from the baseline value, measured before anaesthesia induction. Second, the duration of an absolute MAP of < 65 mmHg during anaesthesia. The baseline blood pressure value was defined as the mean of two measurements at least 5 min apart after the patient’s arrival in the operating room. The duration of the relative systolic blood pressure decrease and the absolute MAP decrease was calculated in each case as a cumulative time in minutes. The systolic and diastolic values were recorded every 5 min from the start of the monitoring in the operating room until the transfer to the post-anaesthesia care unit. The MAP was calculated as the diastolic blood pressure plus 1/3 of the systolic blood pressure minus the diastolic blood pressure.

The secondary outcomes comprised the analyses of the durations of the relative systolic blood pressure deviations (decrease and increase) by 20% from the baseline value and the absolute intraoperative MAP decrease to values < 60 mmHg. In addition, we assessed the proportions of the study treatment duration represented by all aforementioned blood pressure deviations.

Further, we assessed the intraoperative durations of bradycardia (defined as heart rate < 60 beats per minute [bpm]), tachycardia (defined as heart rate > 100 bpm), and the relative heart rate deviation (decrease and increase) by 20% from the baseline values. The heart rate was recorded every 5 min from the start of the monitoring in the operating room until the transfer to the post-anaesthesia care unit. The proportion of the study treatment duration represented by all aforementioned heart rate deviations was also analysed.

Other outcome measures included the analysis of the effect of the confounding variables on the patients’ haemodynamics. These confounders comprised the anaesthetic concentrations (recorded every 5 min), the amount of administered norepinephrine and opioids, and anaesthesia depth (measured by bispectral index [BIS] monitoring). Furthermore, we analysed the influence of a pre-existent arterial hypertension on the need for a haemodynamic support with norepinephrine and the influence of the epidural anaesthesia on the applied opioid amount.

### Statistical analysis

Statistical analysis was performed using SPSS software (Version 24, IBM Corporation, Amonk, New York, USA). After testing for normality using the Shapiro Wilk Test, the continuous data were explored using the Mann-Whitney U test and the dichotomous variables were analysed using Fisher’s exact test. A *p*-value of < 0.05 was considered statistically significant. GraphPad PRISM® (Verson 7.0d, GraphPad Software Inc., La Jolla, California, USA) was used for figure creation.

## Results

**Fig. 1:**
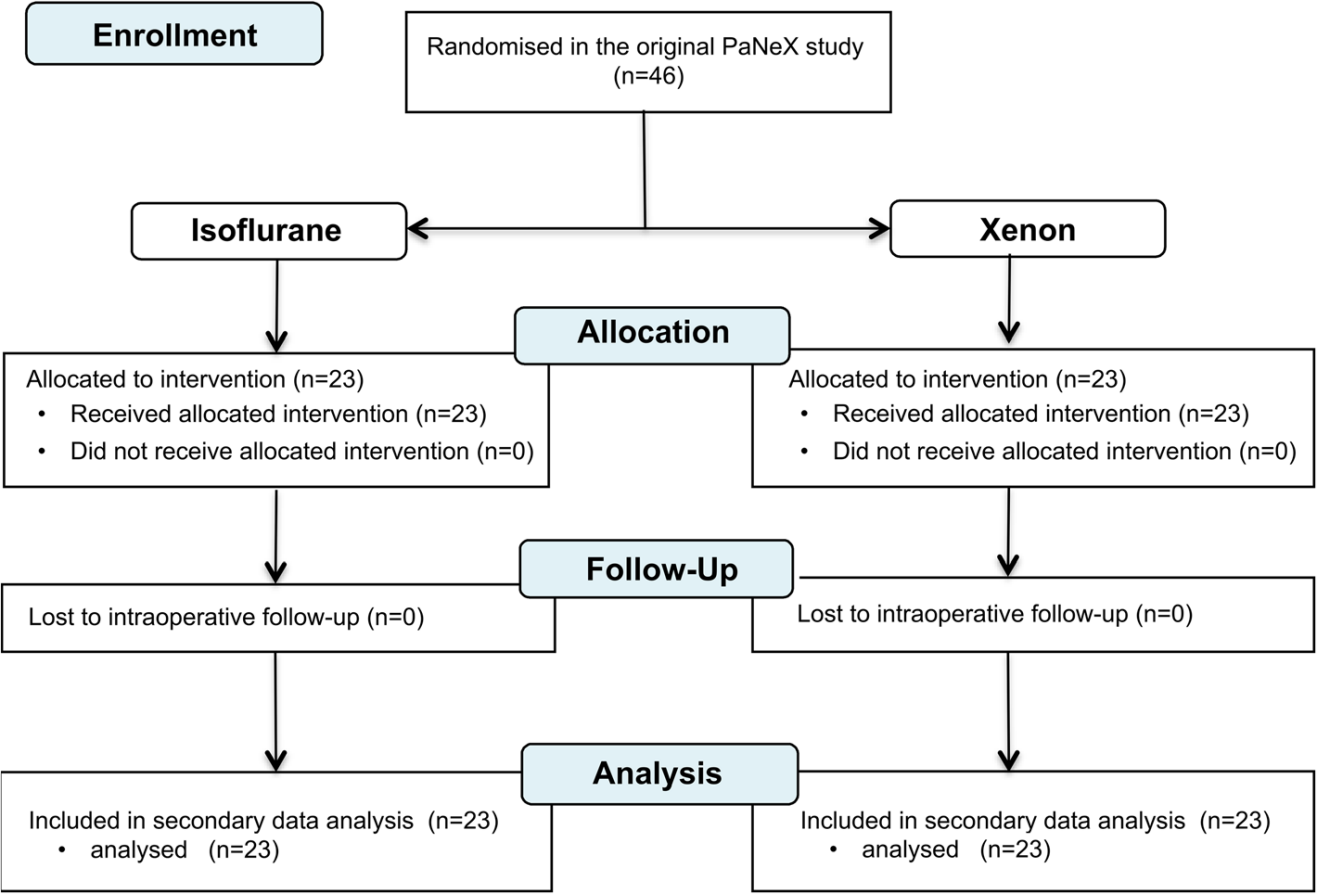
The flowchart^a^ of this secondary analysis ^a^The flowchart was modified according to the PaNeX study for partial nephrectomy with xenon

The results of the main PaNeX study are presented elsewhere [25]. For this secondary analysis, we analysed all the 46 enrolled patients (Fig. 1). Twenty-three patients underwent a xenon and 23 patients underwent an isoflurane anaesthesia. Thirty-two patients were male with a mean (standard deviation [SD]) age of 60 [14] years and mainly ASA II status (*n* = 31). Both study groups were comparable with regard to the sex, age, weight, and the pre-existing chronic diseases [25]. The baseline characteristics are presented in Additional file 1.

### Analysis of the primary outcome

The cumulative duration of the relative systolic blood pressure decrease by > 40% did not significantly differ between the groups; the medians [interquartile range (IQR)] were 5.0 [0–35.0] and 2.5 [0–12.5] minutes for isoflurane and xenon, respectively, *P* = 0.51 (Table 1). In contrast, the cumulative duration of the absolute MAP of < 65 mmHg was significantly longer in the isoflurane than in the xenon group; the medians [IQR] were 25.0 [10.0–47.5] and 0 [0–10.0] minutes for isoflurane and xenon, respectively, *P* < 0.01 (Table 1).

**Table 1:** Blood pressure analyses

	Isoflurane (*n* = 23)	Xenon (*n* = 23)	*P*-value
Blood pressure, median (IQR), mm Hg			
Systolic blood pressure	105.7 [100.9–113.0]	114.2 [104.4–131.3]	0.020*
Diastolic blood pressure	64.0 [56.9–68.5]	75.1 [63.9–83.2]	0.011*
Duration of systolic blood pressure deviation, median (IQR), minutes			
Decrease > 40% from baseline	5.0 [0–35.0]	2.5 [0–12.5]	0.510
Decrease > 20% from baseline	110.0 [47.5–162.5]	60.0 [12.5–120.0]	0.018*
Increase > 20% from baseline	0 [0–2.5]	0 [0–15.0]	0.049*
Proportion of the study treatment duration represented by the systolic blood pressure deviation duration, median (IQR), %			
Decrease > 40% from baseline	2 [0–18.0]	0 [0–7.7]	0.549
Decrease > 20% from baseline	76.9 [26.5–90.9]	33.3 [6.7–77.5]	0.170
Increase > 20% from baseline	0 [0–0.9]	0 [0–6.6]	0.036*
MAP, median (IQR), mmHg	77.2 [72.2–85.1]	84.2 [75.8–100.9]	0.029*
Duration of MAP decrease, median (IQR), minutes			
MAP < 65 mmHg	25.0 [10.0–47.5]	0 [0–10.0]	< 0.002**
MAP < 60 mmHg	5.0 [0–17.5]	0 [0–5.0]	0.041*
Proportion of the study treatment duration represented by the MAP decrease, median (IQR), %			
MAP < 65 mmHg	16.7 [4.2–22.8]	0 [0–5.8]	< 0.004**
MAP < 60 mmHg	3.9 [0–9.5]	0 [0–3.1]	0.048*

The *P*-values were derived using the Mann-Whitney *U* test. * indicates a significant *P*-value, ** indicates a highly significant *P*-value. *IQR* interquartile range, *MAP* mean arterial pressure, *n* number of patients

### Analysis of the secondary outcomes

#### Further blood pressure analyses (proportions, other thresholds, and sensitivity analyses)

The proportion analysis of the duration of cumulative relative blood pressure decrease by > 40% was similar in the two groups, *P* = 0.55. Nevertheless, the duration of MAP of < 65 mmHg was significantly longer in the isoflurane group, *P* < 0.01 (Table 1).

When analysing the relative blood pressure drops > 20%, a significantly longer interval was found with isoflurane, *P* = 0.018. However, the proportion of the study treatment duration did not show a significant result (*P* = 0.170; Table 1).

The cumulative duration of the relative systolic blood pressure increase by > 20% was significantly longer and showed a higher proportion of the study treatment duration in the xenon group with *P* = 0.049 and *P* = 0.036, respectively (Table 1).

The duration of the MAP decrease to values < 60 mmHg was significantly longer in the isoflurane group for both analyses, the cumulative duration and the proportion of the study treatment duration, with *P* = 0.041 and *P* = 0.048, respectively (Table 1).

In an additional sensitivity analysis, we compared the group differences for both MAP and systolic blood pressure, using two mixed-models (repeated measurements ANOVA). For each model, the results showed a significant difference between the groups (*P* < 0.001 each). The overall time effect was also significant (*P* < 0.001 each) for both models (MAP and systolic blood pressure). In contrast, the group-by-time interaction effect was not significant for the MAP (*P* = 0.546), neither for the systolic blood pressure (*P* = 0.183).

Interestingly, though our institutional SOP demanded the maintenance of a MAP ≥65 mmHg during surgery, hypotensive periods were registered in both groups.

#### Heart rate analysis

**Fig. 2:**
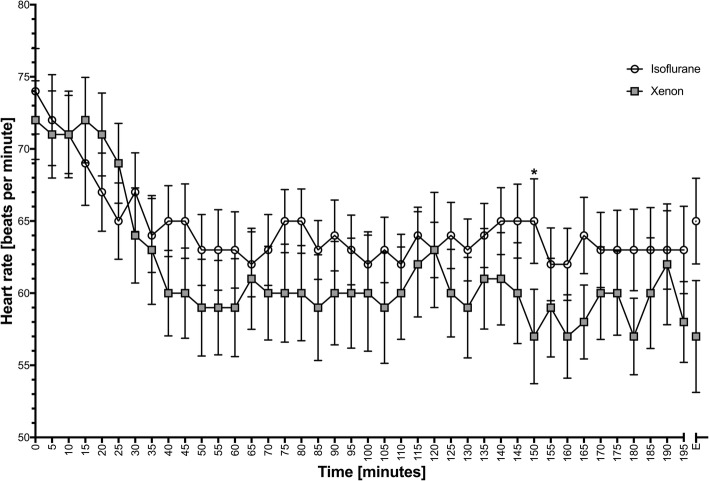
Intraoperative heart rate Intraoperative heart rate in the xenon and isoflurane groups at different time points. The data were analysed using the Mann-Whitney *U* test and are presented as means ± standard deviation. *P-value < 0.05

The mean (SD) intraoperative heart rates were 65.2 (9.1) bpm and 61.6 (13.2) bpm in the isoflurane and xenon group, respectively [25]. Although the patients under xenon anaesthesia showed a lower intraoperative heart rate most of the time (Fig. 2), this difference was not significant (*P* = 0.116) (Table 2). The proportion of the study treatment duration represented by the duration of bradycardia (heart rate < 60 bpm) was also similar in both groups *P* = 0.086 (Table 2).

**Table 2:** Heart rate analyses

	Isoflurane (*n* = 23)	Xenon (*n* = 23)	*P*-value
Heart rate			
Pre-existent bradycardia (heart rate < 60 min^− 1^) during study treatment, n (%)	3 (13)	3 (13)	
Heart rate, mean (SD), beats per minute	65.2 (9.1)	61.6 (13.2)	0.116
Duration of heart rate decrease > 20% from baseline, median (IQR), minutes	35.0 [2.5–77.5]	70.0 [7.5–157.5]	0.163
Proportion of the study treatment duration represented by the duration of a heart rate decrease > 20% from baseline, median (IQR), %	14.6 [0.9–46.0]	69.4 [5.7–88.9]	0.129
Duration of a heart rate increase > 20% from baseline, median (IQR), minutes	0 [0–7.5]	0 [0–12.5]	0.741
Proportion of the study treatment duration represented by the duration of a heart rate increase > 20% from baseline, median (IQR), %	0 [0–4.9]	0 [0–7.4]	0.694
Duration of bradycardia (heart rate < 60 min^−1^), median (IQR), minutes	60.0 [10.0–145.0]	100.0 [47.5–155.0]	0.301
Proportion of the study treatment duration represented by the duration of bradycardia (heart rate < 60 min^−1^), median (IQR), %	32.1 [6.5–79.3]	82.1 [35.4–94.9]	0.086
Pre-existent tachycardia (heart rate > 100 min^− 1^), n (%)	2 (9)	1 (4)	
Duration of tachycardia (heart rate > 100 min^−1^) during study treatment, median (IQR), minutes	0 [0–0]	0 [0–0]	0.415
Proportion of the study treatment duration represented by the tachycardia (heart rate > 100 min^− 1^) duration, median (IQR), %	0 [0–0]	0 [0–0]	0.415

The *P*-values were derived using the Mann-Whitney *U* test. *IQR* interquartile range, *n* number of patients, *SD* standard deviation

Moreover, the episodes of tachycardia, as well as the cumulative duration of a relative decrease or increase of the heart rate by > 20%, were similar in the two groups (Table 2).

An additional sensitivity analysis of the heart rate using a mixed-model repeated measures ANOVA did not show a statistically significant interaction between the time and groups (*P* = 0.897). Furthermore, there was neither a significant main effect for time nor for the group, with *P* = 0.250 and *P* = 0.447, respectively.

#### Norepinephrine and opioid analysis

**Fig. 3:**
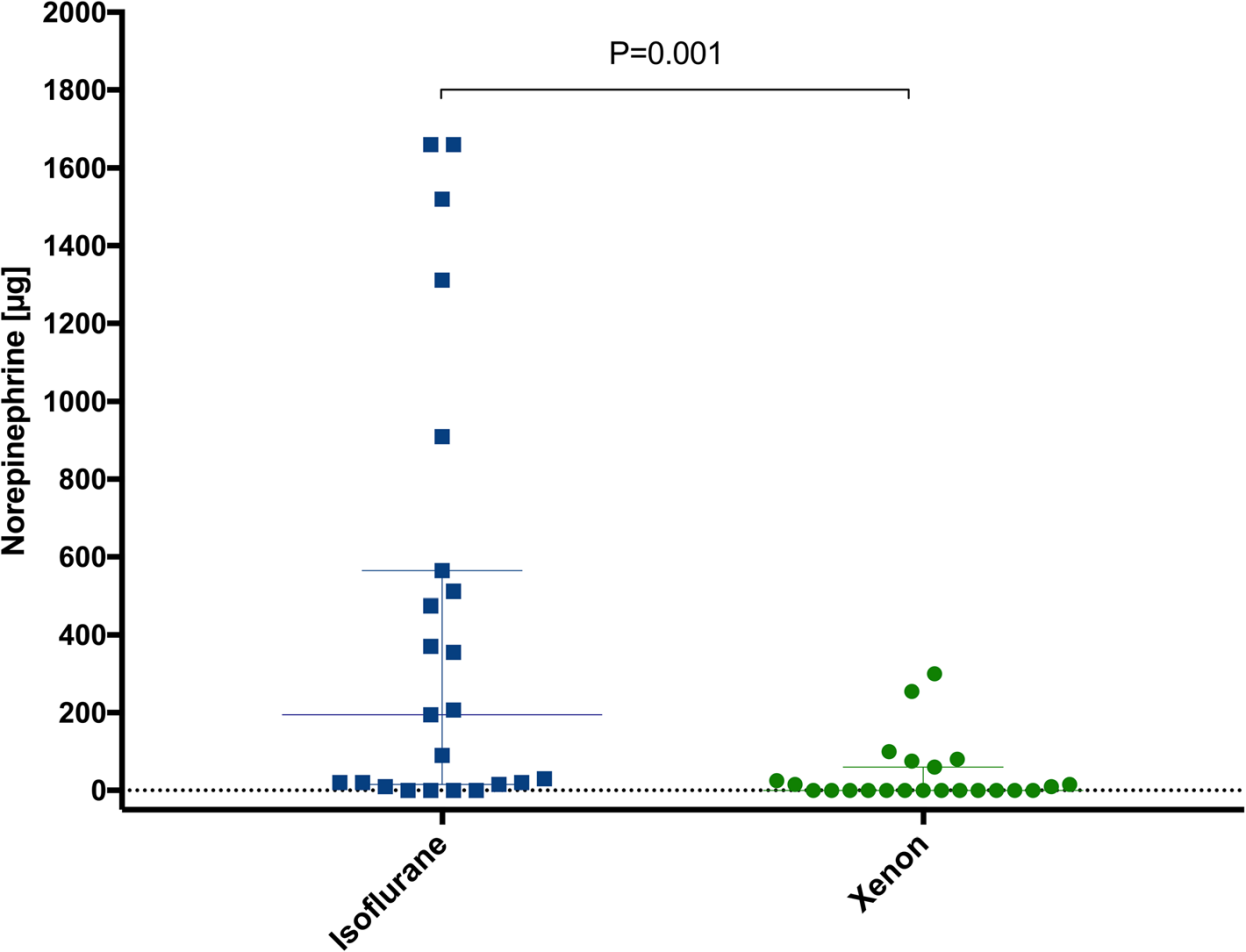
Intraoperative norepinephrine consumption Cumulative intraoperative norepinephrine consumption in the xenon and isoflurane groups, presented as median with interquartile ranges. Individual data are presented as closed circles and squares. The data were analysed using the Mann-Whitney *U* test

The cumulative amount of administered norepinephrine was significantly higher in the isoflurane than in the xenon group, *P* = 0.001 (Table 3). The patients received a median [IQR] norepinephrine dose of 195.0 [17.5–538.5] μg and 0 [0–42.5] μg in the isoflurane and xenon group, respectively (Fig. 3). This significance was more pronounced in the patients with pre-existent arterial hypertension than in those without it, *P* = 0.006 and *P* = 0.040, respectively (Table 3). In addition, the treatment duration with norepinephrine was significantly longer in the isoflurane group (Table 3).

**Table 3:** Norepinephrine and opioid analyses

	Isoflurane (*n* = 23)	Xenon (*n* = 23)	*P*-value
Norepinephrine consumption			
Pre-existent arterial hypertension, n (%)	12 (52)	12 (52)	–
Total amount per group, μg	9945.5	934.5	–
- with pre-existent hypertension	7785.5	644.5	–
- without pre-existent hypertension	2160.0	290.0	–
Cumulative intraoperative consumption, median (IQR), μg	195.0 [17.5–538.5]	0 [0–42.5]	0.001**
- with pre-existent hypertension	422.8 [25.0–1415.5]	15 [0–77.5]	0.006**
- without pre-existent hypertension	20.0 [5.0–275.0]	0 [0–5.0]	0.040*
Duration of norepinephrine administration, median (IQR), min	95.0 [10–192.5]	0 [0–30.0]	0.002**
- with pre-existent hypertension	167.5 [15.0–260.0]	10.0 [0–42.5]	0.008**
- without pre-existent hypertension	20.0 [2.5–120.0]	0 [0–5.0]	0.065
Proportion of the study treatment duration represented by the norepinephrine administration duration, median (IQR), %	57.1 [6.2–91.7]	0 [0–13.2]	0.001**
- with pre-existent hypertension	68.6 [9.4–93.3]	6.7 [0–26.1]	0.008**
- without pre-existent hypertension	15.0 [1.0–88.0]	0 [0–2.6]	0.034*
Opioid (sufentanil) consumption			
Total intraoperative consumption per group, μg	905	1005	
Additional epidural anaesthesia, n (%)	15 (66)	19 (83)	
Cumulative intraoperative consumption, median (IQR), μg	40.0 [30.0–50.0]	30.0 [25.0–55.0]	0.912
- with epidural anaesthesia	30.0 [25.0–47.5]	30.0 [25.0–45.0]	0.891
- without epidural anaesthesia	47.5 [40.0–55.0]	62.5 [50.0–82.5]	0.109

The *P*-values were derived using the Mann-Whitney *U* test. * indicates a significant *P*-value, ** indicates a highly significant *P*-value. *IQR* interquartile range, *n* number of patients

The cumulative amount of sufentanil did not differ between the two groups, *P* = 0.912. An additional epidural anaesthesia was performed in 15 (66%) and 19 (83%) patients, in the isoflurane and xenon group, respectively (Table 3). Epidural anaesthesia led to less opioid consumption, but without significant differences between the groups (Table 3).

#### Confounding factors on haemodynamics

This secondary analysis showed that the proportion of patients with pre-existent hypertension, bradycardia, and tachycardia did not differ between the groups (Tables 2 and 3). However, the analysis of concomitant preoperative antihypertensive medication in the patients with pre-existent hypertension revealed a difference with regard to the number of patients who received angiotensin-converting-enzyme (ACE) or AT_1_ receptor (AR) antagonist before surgery (xenon (11 of 12), isoflurane (7 of 12)). The ACE/AR antagonist therapy was discontinued for all patients 24 h before the surgery in accordance with our SOP.

**Fig. 4:**
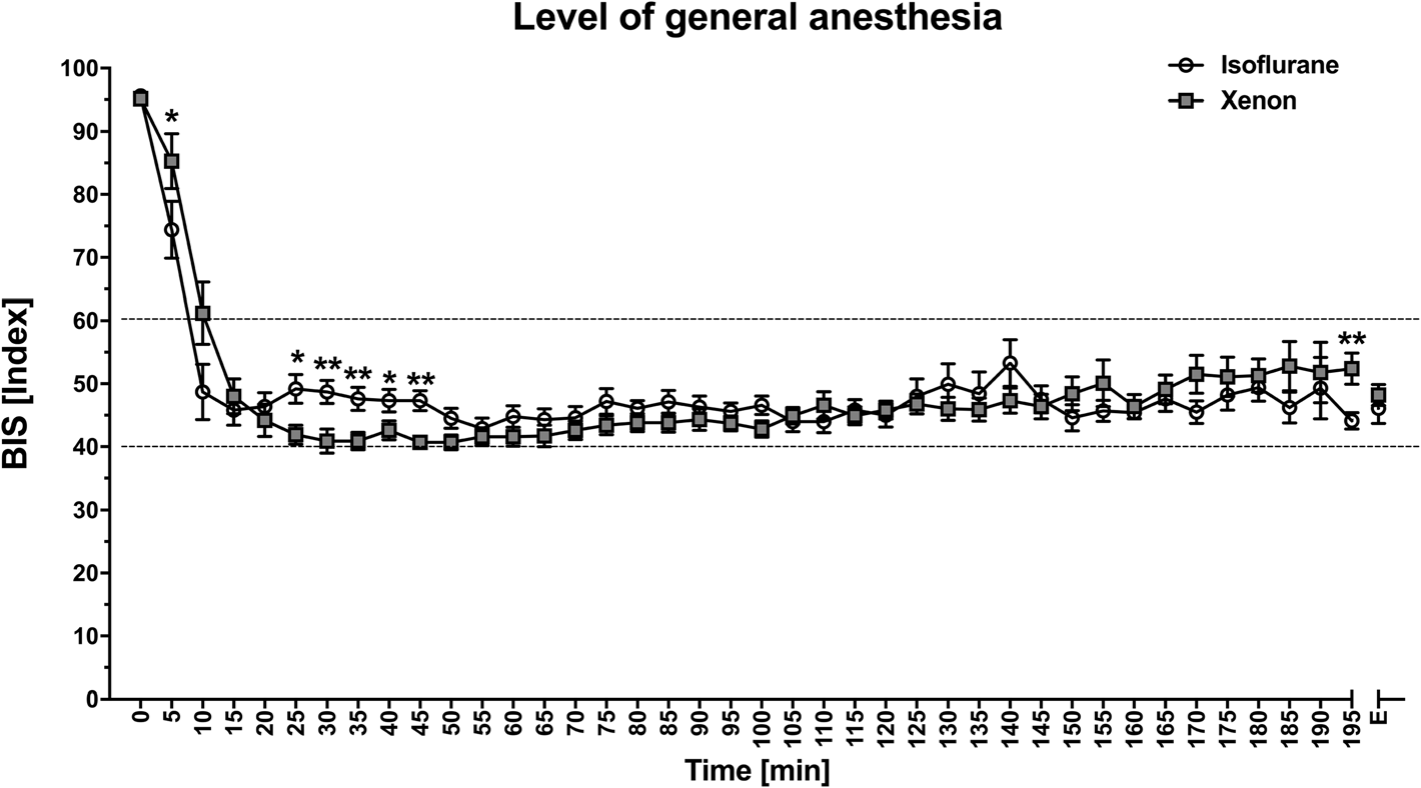
Level of anaesthesia Level of anaesthesia measured by BIS monitoring in the xenon and isoflurane groups at different time points. BIS, bispectral index. The data were analysed using the Mann-Whitney *U* test and are presented as means ± standard deviation. *P-value < 0.05; **P-value < 0.01

**Fig. 5:**
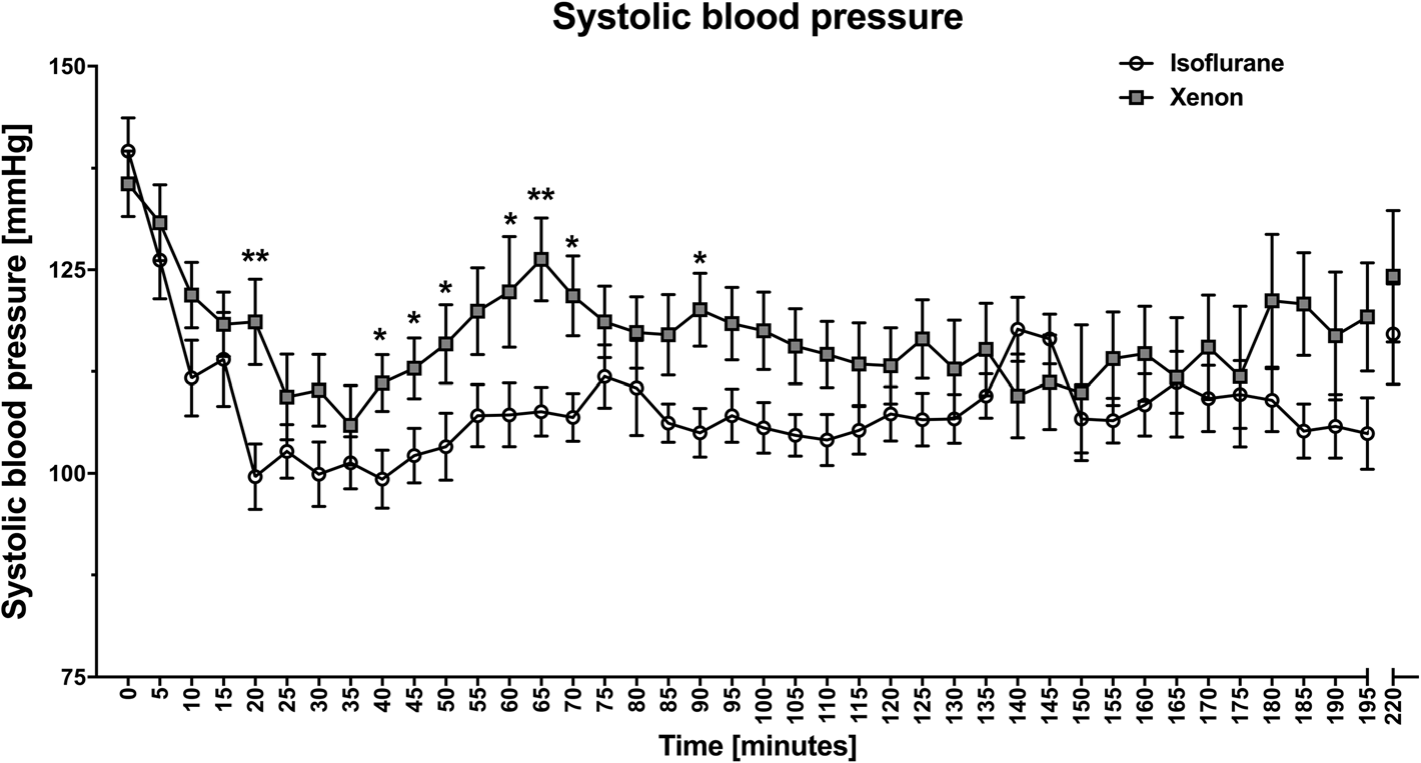
Intraoperative systolic blood pressure Systolic blood pressure in the xenon and isoflurane groups at different time points. The data were analysed using the Mann-Whitney *U* test and are presented as means ± standard deviation. *P-value < 0.05; **P-value < 0.01

Furthermore, there were no differences with regard to the surgery duration, the surgical positioning, the duration of kidney manipulation and ischaemia time, and the histological and pathological analyses (resected tissue volume, weight, and tumour size) between the groups. Four patients in the isoflurane and one patient in the xenon group underwent a total nephrectomy due to an unforeseen intraoperative decision, based on the tumour extent and the urologic SOP. The mean (SD) study treatment duration was 197 (60) minutes in the isoflurane and 166 (59) minutes in the xenon group. The groups showed a similar fluid balance; crystalloid and colloid infusions, and total blood loss showed no significant differences with *P* = 0.702, *P* = 0,248, and *P* = 0.361, respectively. Volatile anaesthetics were administered according to the MAC values in the clinical routine and quantified by BIS. The patients received a mean (SD) of 0.8 (0.1) Vol.%, 0.7 MAC isoflurane, and 51.1 (2.5) Vol.%, 0.8 MAC xenon. The BIS values were comparable (Fig. 4). Both the systolic (Fig. 5) and the diastolic blood pressure were lower in the isoflurane group, *P* = 0.02 and *P* = 0.01, respectively (Table 1) [25]. The important outcomes are listed in the table in the Additional file 2.

## Discussion

The present secondary analysis of the PaNeX study evaluated the haemodynamics during xenon anaesthesia in patients undergoing partial nephrectomy. The xenon group showed a significantly better intraoperative haemodynamic stability, with a lesser need for norepinephrine and no major differences in the heart rates, compared to the isoflurane group.

In contrast to our findings, previous studies have reported an absent or minimal negative impact of xenon on the peripheral vascular resistance or cardiac inotropy [26–28]. Isoflurane was found to exert a cardiodepressant effect [28] and peripheral vascular vasodilatation with consecutive hypotension [28, 29]. Haemodynamic stability was substantially preventing direct renal hypoperfusion and indirect vasoconstriction via neurohumoral responses to the intraoperative hypotension [3].

There is still no consensus regarding the lowest acceptable absolute or relative intraoperative blood pressure threshold and its tolerable maximum duration [8, 12]. The postulated associations of the intraoperative hypotension with adverse events have to be evaluated carefully regarding the thresholds and durations of hypotensive episodes [6, 12]. The common thresholds for systolic blood pressures are described as < 80 mmHg, a MAP < 60 mmHg, or a relative decrease by 30–50% [30]. Our analyses were based on the target blood pressure recommendations of the German DGAI guideline [13]. Although we could not determine a significant difference in the duration of a systolic blood pressure decrease of 40%, the results for a MAP decrease were unequivocal and favoured the xenon anaesthesia. This preservation of high MAP values under xenon anaesthesia might be a crucial factor for prevention of postoperative renal failure [4, 7, 9, 31]. Nevertheless, the thresholds have to be considered individually, since patients with chronic arterial hypertension and altered vascular autoregulation are susceptible to lower blood pressure limits [4, 8, 32]. The significantly larger need for norepinephrine in our patients with pre-existing arterial hypertension compared to those without arterial hypertension supports this assumption. It is remarkable that the patients with arterial hypertension in the isoflurane group needed a significantly higher and prolonged norepinephrine administration than those in the xenon group. One reason might be the vegetative property of xenon. It was shown that the endogen norepinephrine concentration is increased during xenon anaesthesia, without an alteration of the sympathetic activity and baroreflex gain [33]. A norepinephrine re-uptake inhibition in the neuronal cells was postulated [33]. Similar results were found in several large-scale studies [34, 35]. As we have not analysed the plasma concentrations of norepinephrine in our PaNeX study [25], a verification of this assumption is not possible. However, during cardiac surgery, iatrogenic-induced catecholamines were found to be independent predictors of postoperative morbidity and mortality [36] and they are also a risk for the renal function [3]. To the best of our knowledge, it is unknown whether this correlation also applies to the increased levels of the endogen catecholamines. Even though it is recommended to continue the ACE/AR antagonist therapy on the day of a non-cardiac surgery [37], the recent VISION study showed a lower risk for intraoperative hypotension in the patients who withheld their ACE/AR antagonists therapy 24 h before surgery [38]. In accordance with this publication and several other anaesthesia groups [38, 39], our policy was to discontinue the ACE/AR antagonists 24 h before surgery. Therefore, we assume that the different number of patients receiving ACE/AR antagonists more than 24 h before surgery did not have an effect on our results.

Several confounders on the patients’ haemodynamics were analysed in detail and showed no difference in their appearance between the groups. Hypovolaemia is known to be an important additional cause for postoperative renal failure [3, 4]; however, the fluid management did not differ between the groups [25]. It is also unlikely that the similar demographic variables and pre-existent comorbidities may explain the different results.

The usually applied lateral position of the patient during open partial nephrectomy might induce haemodynamic and respiratory changes, but there were no differences regarding the positioning. The anaesthetics exposure times, and the surgical technique, duration and team were similar in our study.

Renal hypoperfusion is known to activate the renin-angiotensin-aldosterone system (RAAS) with a consecutive sympathetic response [2]. About half of the patients in both groups (9 of the 23 in the isoflurane and 10 of the 23 in the xenon group) underwent an intraoperative hilar clamping of similar durations [25]. Thus, the activation of RAAS cannot be the main reason for the haemodynamic differences.

This secondary analysis included all enrolled patients, irrespective of a successful conduction of the partial nephrectomy. Even though there were no statistical differences between the groups regarding the pathological results, there was a difference in the number of patients with an unexpected total nephrectomy. The data on the effects of the unilateral nephrectomy on the levels of blood pressure and RAAS showed no difference before and after the nephrectomy [40]. Therefore, we assume that the blood pressure differences were not influenced by the cases of nephrectomy.

Since most of the anaesthetics are cardiodepressants, the anaesthesia depth might influence the intraoperative haemodynamics. In both groups, the anaesthesia was performed according to the SOPs. The MAC as well as the BIS values were in the recommended range, therefore a comparable anaesthesia depth could be expected. The higher blood pressure in the xenon group might indicate an insufficient analgesia, which induces sympathetic stimulation with tachycardia and hypertension. However, the tendency for lower heart rates argues against this postulation. The analgesic properties of xenon are controversial [41]. Xenon may induce a kind of pain tolerance, which clinically presents as reduced need for opioids [26, 28, 35, 42]. Beside the direct antinociceptive effects via inhibition of the N-methyl-D-aspartate receptors, indirect effects caused by an increased norepinephrine levels in cerebral cortex are considered as explanations [42]. However, the present analysis did not show significantly different consumption of sufentanil, even in combination with the similarly frequent applied epidural anaesthesia, which is known to increase the risk of hypotension [8].

The heart rate is one further important factor on the haemodynamic stability. Though not significant, we confirmed the observations of more pronounced intraoperative heart rate decrease with xenon [26, 33–35, 41, 43–45]. Xenon was described in this context to show a vagotonic effect [46]. In contrast, the heart rates under isoflurane anaesthesia are mainly reported as remaining unchanged [26, 27, 29, 46], whereas our analysis showed also a heart rate decrease. The reason for this observation remains unclear.

What renders our study novel, however, is that we used xenon in a partial nephrectomy setting and had already predefined this secondary analysis. Therefore, we could analyse the prospectively collected data for all patients and we possessed clearly documented baseline blood pressure values. This enabled us to quantify the haemodynamic stability directly via calculation of the cumulative times below different blood pressure thresholds as well as individual relative blood pressure drops from baseline. This is in contrast to other studies, which have only indirectly analysed the haemodynamics via the catecholamine consumption as a surrogate indicator [36, 47].

### Limitations

We acknowledge several limitations in this secondary analysis:

- First, the blood pressure was not measured by invasive methods but non-invasively every five minutes, which likely had an influence on our results [6].
- Second, the attending anaesthetists could not be blinded for safety reasons. Due to the nature of our study, norepinephrine was applied for hypotensive therapy after an inadequate respond to fluid administration, according to the clinical routine in our hospital. The anaesthetists had to adhere to our SOPs for partial nephrectomy. Nevertheless, it might reflect a “real world” situation [48], that the required MAP ≥65 mmHg was not achieved throughout the surgeries.
- Third, this study was a secondary analysis of a prospective trial with the primary outcome *maximum postoperative GFR decrease*. It was not powered to analyse the blood pressure differences.
- Fourth, it may be questionable whether the reduced need for vasopressors and more stable intraoperative haemodynamic with xenon represents a better preservation of renal function apart from facilitating the haemodynamic management. The PaNeX study could only show a tendency to better postoperative renal function with xenon [25]. The underlying reason might be the drop-out of 5 patients from the per protocol analysis or the prevalence of mainly ASA II patients without pre-existent renal disease. It is known that patients with a higher ASA status and preoperatively impaired renal function have a greater risk for perioperative acute renal failure, in particular after an intraoperative hypotension [4, 7]. Nevertheless, we assume that xenon anaesthesia might have a significant impact on the renal outcome in these higher-risk patients. Of note, the enormous price difference between the xenon and isoflurane anaesthesia should always be weighed against the potential benefit of xenon anaesthesia. With approximately 20€ per litre xenon and 35€ per 250 ml isoflurane, we had mean (SD) costs in the xenon and isoflurane group of 196 [37] € and 0.77 (0.27) € per anaesthesia hour, respectively.

## Conclusions

The patients undergoing partial nephrectomy under xenon anaesthesia presented a more stable haemodynamic profile, with a significantly lesser need for norepinephrine than those under isoflurane anaesthesia. The tendency of a better-preserved renal function after partial nephrectomy under xenon anaesthesia might be explained by a better renal perfusion.

## Additional files

Additional file 1: Patients’ baseline characteristics. Modified table according to [25]. ^a^*P*-values were derived using Fisher’s exact test (qualitative data) or the Mann-Whitney U-test (quantitative data). The data are presented as median (interquartile range) or number (proportion). ASA, American Society of Anesthesiologists; COPD, chronic obstructive pulmonary disease; GFR, glomerular filtration rate; IDDM, insulin dependent diabetes mellitus; n, number; NIDDM, non-insulin dependent diabetes mellitus; NYHA, New York Heart Association; PAOD, peripheral artery occlusive disease. (DOCX 94 kb)

Additional file 2: Important outcomes. Modified previously published data [25]. ^a^*P*-values were derived using Fisher’s exact test (qualitative data) or the Mann-Whitney U-test (quantitative data). The data are presented as median (interquartile range) or number and percentage. AKIN, Acute Kidney Injury Network; GFR, Glomerular filtration rate; Min, minutes; n, number; PBRC, packed red blood cells; y/n, yes/ no. (DOCX 22 kb)

## Data Availability

The datasets used and analysed during the current study are available from the corresponding author on reasonable request.

## Abbreviations

ACE: Angiotensin-converting-enzyme
AR: AT_1_ receptor
ASA: American Society of Anesthesiologists
BIS: Bispectral index
bpm: Beats per minute
DGAI: German Society for Anaesthesiology and Intensive Medicine
GFR: Glomerular filtration rate
IQR: Interquartile range
MAP: Mean arterial pressure
n: Number of patients
SD: Standard deviation
SOP: Standard operating procedure
