# Supplementary material for: Better haemodynamic stability under xenon anaesthesia than under isoflurane anaesthesia during partial nephrectomy – a secondary analysis of a randomised controlled trial
Source: BMC Anesthesiol. 2019 Jul 9;19:125. doi: 10.1186/s12871-019-0799-2 (PMC6617591; doi:10.1186/s12871-019-0799-2)
Supplement: Supplementary file 1 — Patients’ baseline characteristics. Modified table according to [25]. aP-values were derived using Fisher’s exact test (qualitative data) or the Mann-Whitney U-test (quantitative data). The data are presented as median (interquartile range) or number (proportion). ASA, American Society of Anesthesiologists; COPD, chronic obstructive pulmonary disease; GFR, glomerular filtration rate; IDDM, insulin dependent diabetes mellitus; n, number; NIDDM, non-insulin dependent diabetes mellitus; NYHA, New York Heart Association; PAOD, peripheral artery occlusive disease. (DOCX 94 kb) [file 12871_2019_799_MOESM1_ESM.docx]

**Additional file 1. Patient baseline characteristics.**

| **Group** | **Total (n=46)** | **Isoflurane (n=23)** | **Xenon (n=23)** | ***P-*value**^a^ |
| --- | --- | --- | --- | --- |
| Sex: male, n (%) | 32 (69.9) | 15 (65.2) | 17 (73.9) | 0.749 |
| Age [yrs.] | 59.5 [52-71] | 61 [48-81] | 59 [52-72] | 0.912 |
| Height [cm] | 173 [168-180] | 175 [168-180] | 172 [166-180] | 0.991 |
| Weight [kg] | 82 [69-93] | 82 [67-93] | 82 [70-93] | 0.834 |
| ASA I/II/III, n (%) | 4/31/11 (8.7/67.4/23.9) | 2/17/4 (8.7/73.9/17.4) | 2/14/7 8.7/60.9/30.4) | 0.663 |
| Chronic heart failure (NYHA II), n (%) | 7 (15.2) | 3 (13.0) | 4 (17.4) | 1.0 |
| Ischemic heart disease, n (%) | 2 (4.3) | 1 (4.3) | 1 (4.3) | 1.0 |
| Myocardial Infarction, n (%) | 2 (4.3) | 1 (4.3) | 1 (4.3) | 1.0 |
| COPD/Asthma, n (%) | 0 | 0 | 0 | 1.0 |
| NIDDM/IDDM, n (%) | 7 (15.2)/1 (2.2) | 3 (6.5)/0 | 4 (17.4)/1 (4.3) | 0.699 |
| Renal impairment, n (%) | 0 | 0 | 0 | 1.0 |
| Baseline GFR [ml min-1 1,73 cm-²] | 87.9 [79.4-98.2] | 85.6 [81.4-97.4] | 88.0 [74.5-100.9] | 0.668 |
| Arterial hypertension, n (%) | 24 (52.2) | 12 (52.2) | 12 (52.2) | 1.0 |
| Stroke, n (%) | 1 (2.2) | 1 (4.3) | 0 | 1.0 |
| PAOD, n (%) | 0 | 0 | 0 | 1.0 |

Modified table according to (25).

^a^*P*-values are from Fisher's exact test (qualitative data) or Mann-Whitney U-test (quantitative data), respectively. Data are presented as median (interquartile range) or number (proportion).

ASA, American Society of Anesthesiologists; COPD, chronic obstructive pulmonary disease; GFR, glomerular filtration rate; IDDM, insulin dependent diabetes mellitus; n, number; NIDDM, non-insulin dependent diabetes mellitus; NYHA, New York Heart Association; PAOD, peripheral artery occlusive disease
