# Supplementary material for: Better haemodynamic stability under xenon anaesthesia than under isoflurane anaesthesia during partial nephrectomy – a secondary analysis of a randomised controlled trial
Source: BMC Anesthesiol. 2019 Jul 9;19:125. doi: 10.1186/s12871-019-0799-2 (PMC6617591; doi:10.1186/s12871-019-0799-2)
Supplement: Supplementary file 2 — Important outcomes. Modified previously published data [25]. aP-values were derived using Fisher’s exact test (qualitative data) or the Mann-Whitney U-test (quantitative data). The data are presented as median (interquartile range) or number and percentage. AKIN, Acute Kidney Injury Network; GFR, Glomerular filtration rate; Min, minutes; n, number; PBRC, packed red blood cells; y/n, yes/ no. (DOCX 22 kb) [file 12871_2019_799_MOESM2_ESM.docx]

**Additional File 2. Important outcomes**

| **Group** | **Total (n=46)** | **Isoflurane (n=23)** | **Xenon (n=23)** | ***P-*value**^a^ |
| --- | --- | --- | --- | --- |
| **Intraoperatively outcomes** | | | | |
| Anaesthesia expostition time [min] | 171.5 [125-214] | 176 [135-237] | 153 [110-203] | 0.093 |
| Duration of surgical intervention [min] | 139 [95-186] | 139 [95-194] | 149 [82-175] | 0.575 |
| Kidney ischemia/ Hilar clamping time [min] | (n=19) 12 [10-18] | (n=9), 11 [10-13.5] | (n=10), 14.5 [10-19] | 0.315 |
| Total infusion of crystalloids [ml] | 2000 [1500-2500] | 2000 [1500-3000] | 2000 [1500-2500] | 0.702 |
| Total infusion colloids [ml] | 500 [500-500] | 500 [500-1500] | 500 [500-500] | 0.248 |
| Total blood loss [ml] | 210 [100-380] | 150 [150-450] | 220 [80-350] | 0.361 |
| Total urine output [ml] | 150 [80-250] | 120 [50-226] | 170 [100-300] | 0.308 |
| Renal tissue excision volume [cm³] | 53.2 [14.0-184.8] | 61.2 [11.1-184.8] | 46.3 [16.2-247.5] | 0.829 |
| Renal tissue excision weight [g] | 28.9 [7.4-142.0] | 109.0 [8.4-187.0] | 22.5 [7.0-65.9] | 0.122 |
| Tumor size [cm] | 3.6 [2.3-4.8] | 4.1 [2.3-5.9] | 3.4 [2.3-4.5] | 0.510 |
| **Clinical outcomes** | | | | |
| Maximum GFR decrease within 7 postoperative days [ml min^-1^ 1,73 cm^-^²] | -15,2 [-30.8 - -4.5] | -22.1 [-44.8 - -5.0] | -10.8 [-27.5 - 0,1] | 0.073 |
| Adverse events [n] | 1 [1-2] | 2 [1-3] | 1 [0-2] | 0.001 |
| - Intraoperative hypotension. requiring catecholamines, n (%) | 29 (63) | 19 (83) | 10 (44) | 0.013 |
| - Nausea, n (%) | 10 (22) | 7 (30) | 3 (13) | 0.284 |
| - Emesis, n (%) | 3 (7) | 2 (9) | 1 (4) | 1.000 |
| - AKIN 1/2, n (%) | 13/5 (28/11) | 7/4 (30/17) | 6/1 (26/4) | 0.387 |
| - Anemia, requiring PRBC transfusion, n (%) | 4 (8) | 4 (17) | 0 (0) | 0.109 |
| - Post-operative bleeding/ hematoma, n (%) | 4 (9) | 4 (17) | 0 (0) | 0.109 |
| - Surgical Revision, n (%) | 1 (2) | 1 (4) | 0 (0) | 1.000 |
| - Nephrectomy, n (%) | 5 (11) | 4 (17) | 1 (4) | 0.346 |
| Serious adverse event [n] | 0 | 0 | 0 | - |

Modified previously published data (25).

^a^ P-values are from Fisher's exact test (qualitative data) or Mann-Whitney U-test (quantitative data). Data are presented as median (interquartile range) or number and percentage.

AKIN, Acute Kidney Injury Network; GFR, Glomerular filtration rate; Min, minutes; n, number; PBRC, packed red blood cells; y/n, yes/ no.
